# Exploring the use of POLY4 for the improvement of productivity, peanut quality, and soil properties in Southern India

**DOI:** 10.3389/fpls.2024.1448909

**Published:** 2024-10-14

**Authors:** Kodigal A. Gopinath, Gandhamanagenahalli A. Rajanna, Venugopalan Visha Kumari, Vinod Kumar Singh, B. C. Ajay, Neeraj K. Awasthi, Vipin Mishra, Suvana Sukumaran, Govindarajan Venkatesh, Bollam Rajkumar

**Affiliations:** ^1^ ICAR-Central Research Institute for Dryland Agriculture (CRIDA), Hyderabad, India; ^2^ ICAR-Directorate of Groundnut Research, Regional Research Station, Anantapur, India; ^3^ AngloAmerican Crop Nutrients Pvt. Ltd, New Delhi, India

**Keywords:** gypsum, nodules, peanut, pod yield, polyhalite based POLY4, oleic acid

## Abstract

Polyhalite-based POLY4, a multi-nutrient source containing potassium, calcium, magnesium, and sulphur, is increasingly recognised for its potential to improve crop yields and soil health in agricultural systems. It is also been considered as a feasible approach for addressing the deficiency in potassium, calcium, and sulphur within a single application source. The present study aimed to investigate the impact of polyhalite-based POLY4 application, either as a complete or partial substitute for traditional potassium fertiliser and gypsum supplement, on the improvement of peanut (*Arachis hypogaea*) growth and soil quality. An extensive field study was conducted from 2021 and 2022, employing ten distinct nutrient management treatments with three replications in a randomised complete block design. The findings of the study indicated that the application of polyhalite (POLY4) in conjunction with only NP fertilisers resulted in a higher yield advantage (approximately 150–200 kg ha^-1^) than in plots treated with NPK + gypsum (at 500 kg ha^-1^) and control plots. The application of polyhalite-based fertiliser (POLY4) at a rate that was 100% equivalent to K along with NP fertilisers resulted in a significant increase in pod yield (5.3–12.8%) over NPK + gypsum and control plots. Thus, the increased crop yield led to an increase in gross returns of 4.88% and in net returns of 4.28% with the application of POLY4 (100%) + NP fertilisers over other treatments. Likewise, variable rates of conventional fertilisers along with POLY4 (100% recommended) + NP + gypsum at 310 kg/ha significantly increased the linoleic acid content (38.5%), oleic acid content, and oil content (48.1%) by reducing palmitic acid (11.96%) content in the groundnut seed. Interestingly, POLY4 use at the 50% recommended rate also resulted in yields that were comparable with those obtained with 100% NPK. Therefore, applying POLY4, a polyhalite fertiliser, in either a 100% or 50% equivalent of essential K can be an effective way of increasing the production of peanut crops and promoting agricultural sustainability.

## 1 Introduction

Peanuts (*Arachis hypogaea*) are an annual growing legume that have an indeterminate growth habit while producing its fruit (the peanut) under the ground. Peanuts are grown all over the world and are mostly made for their oil and seeds, which are high in protein, fats, and nutrients (Tam et al., 2016; Toomer, 2018; Meena et al., 2022). Peanuts are grown in tropical and subtropical areas (Xue et al., 2021). They are an economically important oilseed, animal feed, and food item. There are 112 countries in Asia, Europe, the Americas, Africa, and Oceania that grow peanuts (Tam et al., 2016; Tan et al., 2022). In 2021–22, there were 32.72 million ha of groundnuts grown around the world, with an average yield of 1.65 Mg ha^-1^ and a production of 53.92 million tonnes (FAO, 2022). Groundnut is an important oilseed crop in India, which contributes 35.29% of the total oilseeds grown. In 2021–22, groundnuts are grown on an area of 5.96 mha in India, with a production of 10.2 mt and a yield of 1.7Mg ha^-1^ (FAO, 2022). Although India has the largest groundnut area and produces the most groundnuts, its output of 1,716 kg ha^-1^ is significantly lower than that of the USA, China, and a few other countries. The main reason for this low output in India is that this crop is mostly grown in rainfed situations (approximately 85% of the area), is vulnerable to weather changes, and is grown in low-fertility and light-textured soils (Gopinath et al., 2022). According to Singh et al. (1997) and Meena et al. (2022), even if groundnuts can withstand drought, they do not receive adequate nutrients, which results in low production. However, there are widespread deficiencies of macro, micro, and secondary nutrients under rainfed conditions, estimated at 89% for N (63% low and 26% medium), 80% for P (42% low and 38% medium), 50% for K (13% low and 37% medium), 41% for S, 48% for Zn, 33% for B, 12% for Fe, 13% for Mo, 5% for Mn, and 3% for Cu (Srinivasarao and Vittal, 2007; Sanyal et al., 2014; Shukla et al., 2021). In recent years, there have been significant observations of magnesium (Mg) and calcium (Ca) deficiencies occurring on a widespread basis in red and lateritic sandy soils (Shukla et al., 2021). Thus, Ca and Mg deficiencies are detrimental to the growth of a wide variety of crops, including peanuts, sunflowers, rainfed rice, fruits, and vegetables (Srinivasarao et al., 2015; Gangothri and Dadhich, 2020; Chandrakala et al., 2021).

Potassium (K) is considered a crucial nutrient for the growth and development of groundnut, ranking second only to phosphorus (P) and nitrogen (N) in terms of importance (Sanyal et al., 2014; Das et al., 2022). Potassium significantly influences yield, drought tolerance, and overall plant health. Numerous studies have documented the favourable impact of potassium (K) application on both the yield and quality of groundnut, particularly in Alfisols characterised by low levels of exchangeable K (Srinivasarao et al., 2007; Sanyal et al., 2014; Srinivasarao et al., 2014; Das et al., 2022; Tan et al., 2022). According to studies by Tam et al. (2016) and Tan et al. (2022), it is necessary to provide peanut plants with sufficient levels of sulphur (S) and potassium (K) to achieve significant yields, whereas nitrogen (N) requirements remain relatively low due to the leguminous nature of the crop. Nevertheless, the implementation of K management practices is contingent upon several factors such as the existing soil K levels, the availability of different sources, the methodologies employed for application, and the specific requirements of the crops being cultivated. Muriate of potash (MOP), also known as potassium chloride (KCl) with a composition of 60% K_2_O, is widely used as a primary source of potassium oxide (K_2_O) in many agricultural contexts (Heba et al., 2021; Meena et al., 2022). This preference is mostly attributed to its relatively affordable cost and widespread accessibility across different crop types and geographical regions. Similarly, the use of polyhalite fertiliser presents a viable approach to mitigating potassium and sulphur deficiencies as the product contains potassium and sulphur.

In addition to conventional fertilisers, alternative multi-nutrient compositions such as polyhalite have been identified as possible resources for nutrient management in groundnut cultivation and for the preservation and restoration of soil fertility (Tan et al., 2022). Polyhalite [K_2_Ca_2_Mg(SO_4_)_4_.2H_2_O] is a naturally occurring mineral that consists of four essential nutrients; 14.0% dipotassium oxide (K_2_O), 17.0% calcium oxide (CaO), 6.0% magnesium oxide (MgO), and 19.0% sulphur (S) (Sirius Minerals, 2016). These macronutrients are crucial for supporting the overall growth and development of crop plants. Polyhalite, a potential fertiliser, exhibits less water solubility than conventional sources, suggesting the possibility of a gradual nutrient release (Barbarick, 1991; Yermiyahu et al., 2017). The various forms of granulated, powdered, or chipped substances enable convenient use across diverse soil conditions. All these nutrients play a crucial role in the metabolic processes of plants and contribute significantly to the growth and development of crops. Polyhalite is a chemically neutral salt that exhibits a solubility of 27 g L^-1^ at a temperature of 25°C. Furthermore, it contains less than 2% chloride (Cl), which renders it appropriate for the cultivation of chloride-sensitive crops, such as potatoes and tobacco (Sirius Minerals, 2016).

There is a limited availability of literature regarding the application of polyhalite fertiliser in the context of peanut and other oilseed crops. Some studies have demonstrated the efficacy of polyhalite as a fertiliser for rice (Huang and Chen, 1990), corn (Wang et al., 2018), wheat (Kumar et al., 2023), oats (Ola et al., 2023) peanut (Tan et al., 2022), potato (Mello et al., 2018a), mustard (Tiwari et al., 2015), tomato (Mello et al., 2018b), and sugarcane (Bhatt et al., 2021; Herrera et al., 2022). Mello et al. (2018b) observed that soils treated with polyhalite fertiliser exhibited elevated levels of Ca and Mg in comparison with soils treated with alternative K fertilisers. This research has shown that using polyhalite as a potassium source instead of more conventional choices like MOP or SOP results in equivalent or higher yields and increased quality. Consequently, POLY4 exhibits the potential for application as a potassium fertiliser in farming operations. On the other hand, pertinent data about POLY4 (polyhalite) and its effect on groundnut crop performance in India is currently lacking. As a result, field research was conducted to investigate the effects of POLY4 on groundnut farming in the semi-arid and desert regions of Telangana and Andhra Pradesh, India. The objective of the research was to assess how POLY4 (polyhalite) differed from other potassium sources in terms of its impact on groundnut yield, quality, soil properties, and the profitability of rainfed peanut cultivation.

## 2 Materials and methods

### 2.1 Study sites and weather data

Field experiments were conducted at two locations, Gungal Research Farm of the ICAR-Central Research Institute for Dryland Agriculture (CRIDA), Hyderabad [17°40’ 40.4” N and 78°39’, 55.7’’ E], and the Regional Research Station of the ICAR-Directorate of Groundnut Research (DGR), Ananthapur, Andhra Pradesh [14.6873403 N and 77.6693692 E], during the rainy seasons (June to October) of 2021 and 2022. The soil texture of the experimental field was red sandy loam, specifically belonging to the Ustic Haplorgids soil type. The initial soil pH of Ananthapur was 6.1, with an organic carbon content of 0.31%, nitrogen concentration of 112 mg kg^-1^, phosphorus concentration of 18 mg kg^-1^, and potassium concentration of 122 mg kg^-1^. At Hyderabad, the soil was sandy loam, moderately acidic in reaction (pH 5.46), low in organic carbon (0.46%), available nitrogen (129.2 kg ha^-1^), and available phosphorus (14.0 kg ha^-1^), medium in available potassium (115.2 kg ha^-1^), and high in calcium, magnesium (5.25, 3.39 kg ha^-1^), and sulphur (14.60 kg ha^-1^). Among the micronutrients, DTPA-extractable zinc was low (0.46 mg kg^-1^), DTPA-extractable iron was medium (8.74 mg kg^-1^), and DTPA-extractable manganese (20.16 mg kg^-1^) and DTPA-extractable copper (1.65 mg kg^-1^) were high in soil.

**Figure 1:**
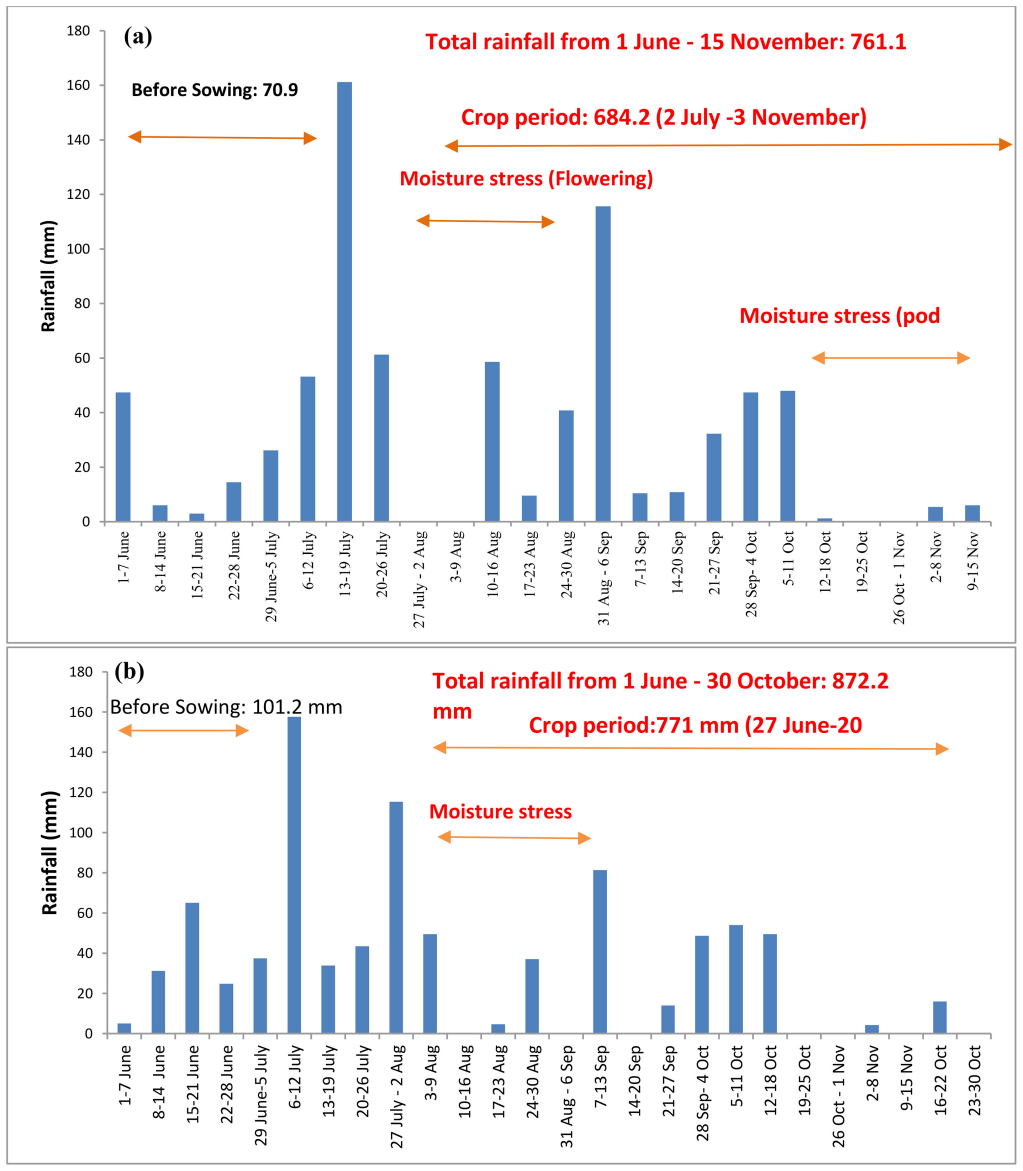
Total rainfall at ICAR-CRIDA, Hyderabad, from **(A)** 1 June to 30 October 2021 and **(B)** 1 June to 30 October 2022.

ICAR-CRIDA, Hyderabad weather: During the crop growing period, the weekly mean maximum and minimum temperature ranged from 22.3°C to 36.0°C and 19.7°C to 25.0°C, respectively (**Supplementary Figure S1**). Morning relative humidity during this period fluctuated between 74.6% and 99.6%, averaging at 94.9%. Afternoon humidity ranged between 35.2% and 80.9%, with an average of 65.6%. Over the crop period, total rainfalls of 684.2 and 771 mm were received in the years 2021 and 2022, respectively (**Figure 1**). In both years, the crop experiences stress during the flowering and pod development stage. The weekly mean bright sunshine hours per day and mean evaporation during the crop growth period ranged from 1.6 to 9.7 h and 3.3 to 7.8 mm day^-1^, respectively.

**Figure 2:**
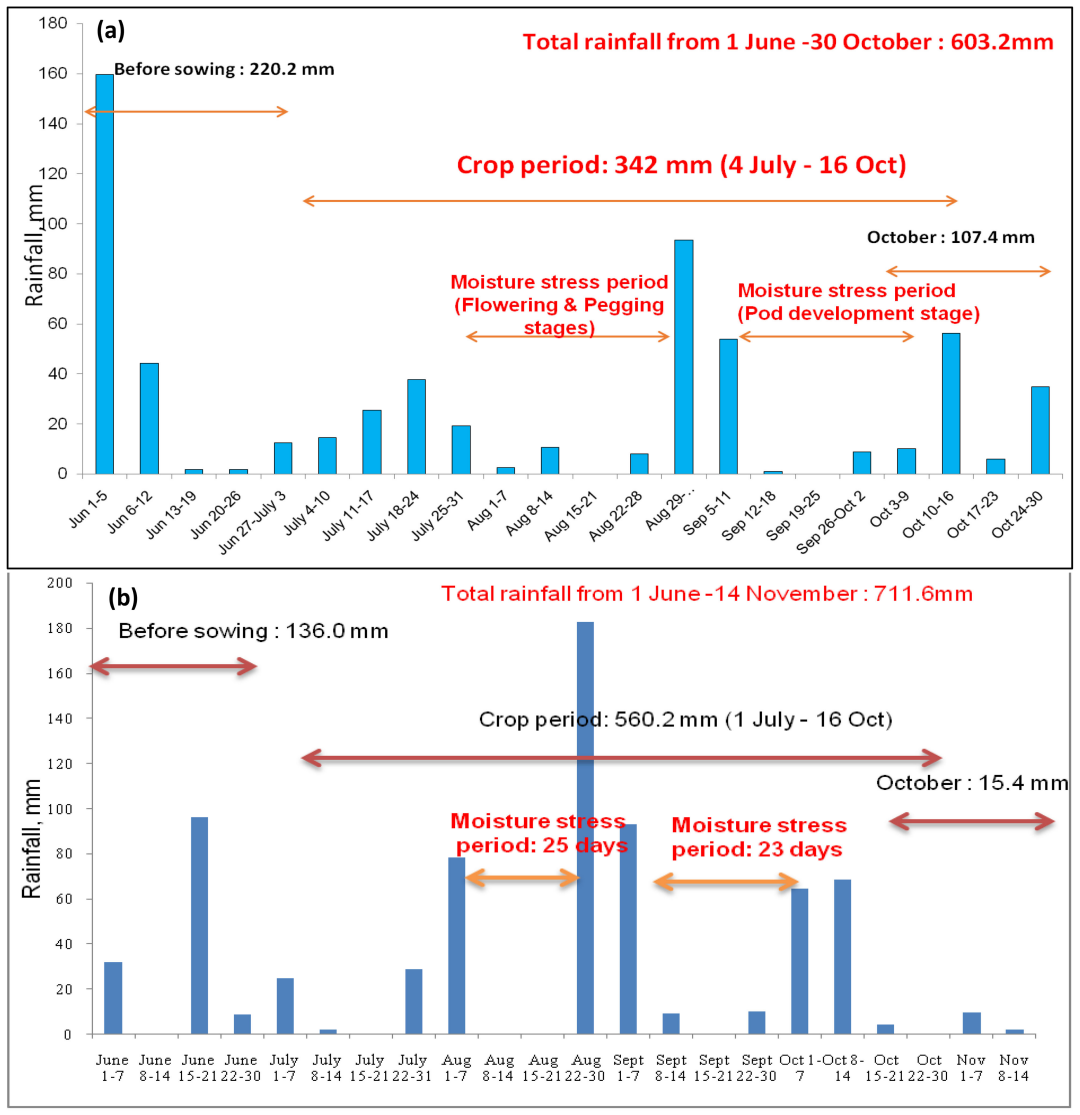
Total rainfall at ICAR-DGR, Ananthapur, from **(A)** 1 June to 30 October 2021 and from **(B)** 1 June to 15 November 2022.

ICAR-DGR, Ananthapur weather: During the experimental period, daily maximum temperatures ranged between 32°C to 34°C in the month of June and July during 2021-22 and 2022-23. Daily minimum air temperature varied from 19–24°C in the month of September and October during both years (**Supplementary Figure S2**). Drought stress was most predominant in the Ananthapur region. Although the crop water requirement of groundnut is approximately 600 mm, the crop received only 342 mm of rainfall during the crop growth period spanning from July to the first fortnight of October 2021 (**Figure 2**). In the year 2022, the region experienced a total rainfall of 560.2 mm (**Figure 2**) during the groundnut crop’s growth stages, significantly influencing the crop’s production potential. During the crop growth period, two major drought stress periods were observed: one at the flowering stage and one at the pod development stage (as depicted in **Figures 1**, **2**).

### 2.2 Experimental details and crop management

The experiment consisted of 10 treatments, each of which was replicated three times in a randomised block design. The treatment details are as follows:

**Table:** 

T_1_	Recommended NPK + gypsum application at 500 kg ha^-1^
T_2_	Recommended NP only
T_3_	Recommended NPK only
T_4_	Recommended NP + MoP 50% only
T_5_	NP+ POLY 4 at 100% recommended dose
T_6_	Recommended NP + POLY 4 at 100% recommended dose + gypsum application at 310 kg ha^-1^
T_7_	Recommended NP + POLY 4 at 50% recommended dose
T_8_	Recommended NP + POLY 4 at 50% recommended dose + gypsum at 310 kg ha^-1^
T_9_	Recommended NP + gypsum at 500 kg ha^-1^
T_10_	Control

The treatment details, along with the corresponding fertiliser application rates, applied to the groundnut crop at both locations in both research seasons, are presented in **Table 1**. The desired fertilisers at both locations were measured as per the recommended rates of 20:40:50 kg NPK ha^-1^ and applied at the time of sowing. The sources of fertilisers were DAP, Urea, MOP, and Polyhalite. The applied fertilisers were thoroughly mixed into the soil, and groundnut K-9 (Hyderabad) and K-6 (Ananthapur) seeds were manually sown in the seed furrows. In Anantapur, the crops were sown on 4 July 2021 and 30 June 2022, and harvested on 16 October 2021 and 17 October 2022 during the respective crop years. In Hyderabad, the crops were sown on 2 July and harvested on 3 November in 2021. In 2022, the crop was sown on 27 June and harvested on 20 October. Seed furrows were opened at 30 × 10 cm row spacing immediately after rainfall and seeds were sown with a plot size of 4.5 m × 6 m = 27.0 m^2^. Gypsum application at 500 kg ha^-1^ was carried out 25 days after sowing when sufficient moisture was present in the soil. At various growing phases, all growth and yield observations were recorded. The crop was threshed plot-wise and the seed yield thus obtained from the net plot was converted into Mg ha^-1^.

**Table 1:** Treatment details and fertiliser rates applied in the respective treatments.

Treatment	Source wise contribution (kg ha^-1^)					Nutrient wise contribution (kg ha^-1^)						
	DAP	Urea	Gypsum	MOP	Poly4	N	P_2_O_5_	K_2_O	S	MgO	CaO	Cl
T1: Recommended NPK + gypsum at 500 kg/ha	87	10	500	83	–	20.3	40.0	50.0	90.0	0.0	161.0	38.3
T2: Recommended NP only	87	10	–	–	–	20.3	40.0	0.0	0.0	0.0	0.0	0.0
T3: Recommended NPK only	87	10	–	83	–	20.3	40.0	49.8	0.0	0.0	0.0	38.2
T4: NP + MOP 50%	87	10	–	41.5	–	20.3	40.0	24.9	0.0	0.0	0.0	19.1
T5: NP + POLY4 at 100% recommended dose	87	10	–	–	357	20.3	40.0	50.0	67.8	21.4	60.7	10.7
T6: NP + POLY4 at 100% recommended dose + gypsum at 310 kg/ha	87	10	310	–	357	20.3	40.0	50.0	123.6	21.4	160.5	10.7
T7: NP + POLY4 at 50% recommended dose	87	10	–	–	178.5	20.3	40.0	25.0	33.9	10.7	30.3	5.4
T8: NP + POLY4 at 50% recommended dose + gypsum at 310 kg/ha	87	10	310	–	178.5	20.3	40.0	25.0	89.7	10.7	160.5	5.4
T9: Recommended NP + gypsum at 500 kg/ha	87	10	500	–	–	20.3	40.0	0.0	90.0	0.0	161.0	0.0
T10: Control	0	0	0	0	0	0	0	0	0	0	0	0

### 2.3 Data collection

At the pod development stage in 2022–23, three representative groundnut plants were dug-out independently from the sampling rows at a depth of 30 cm using a root sampler. The soil mass and plant roots were placed in a fine mesh cloth bag and then submerged in water for an hour to loosen the dirt. After the dirt had settled, the roots of each plant were washed and the number of nodules was recorded. After determining their fresh weight, nodules were placed in the oven and dried at 65°C for 2 days.

The estimation of pod yield and haulm yield was conducted by harvesting entire plants by uprooting them from the net plot area of 4.2 m × 5 m = 21 m^2^. The weights of all pods and kernels obtained from these plants were measured for analysis and subsequently converted into pod yield per hectare. The sun-dried plants were processed by threshing and pods were separated using the shelling method to determine their oil content and nutrient composition. Subsequently, the kernels were weighed to determine their 100-kernel weight. The biological yield was determined by combining the pod yield and haulm yield.

### 2.4 Soil analysis

Soil samples were obtained from the 0–15 cm soil profile using polythene bags from every plot within the experimental field at the beginning and conclusion of each experimental period. The soil samples were subjected to air drying, and the determination of organic carbon content was conducted using the Walkley and Black method (Walkley and Black, 1934). Analysis of accessible nitrogen, phosphorus, and potassium was performed after the materials were air-dried, crushed, and sieved through a 2 mm mesh sieve. The alkaline potassium permanganate (KMnO_4_) method proposed by Subbiah and Asija (1956) was used to calculate the available N, and the results were reported as kg ha^-1^. The amount of available P and K was calculated using Olsen (1954) and Jackson’s (1973) neutral ammonium acetate extraction method and given as kg ha^-1^. Using an atomic absorption spectrophotometer, the amount of available zinc, iron, copper, and manganese in soil extracts was calculated and expressed in mg/kg (Lindsay and Norvell, 1978).

### 2.5 Fatty acid profile estimation

With a Soxhlet device (Extraction unit, Gerhardt-SOXTHERM), seed oil was extracted in hexane. A volume of 2 cc of 13% methanolic KOH was used to transesterify oil for 30 min at 55°C. Hexane was used to extract the organic phase, and water was used to rinse it until the pH was neutral. To create methyl esters, the hexane was dried over anhydrous sodium sulphate and then concentrated using nitrogen. An Agilent 7890B gas chromatograph (Santa Clara, California, USA) fitted with an autosampler and flame ionisation detector (FID) was used to measure the content of fatty acids. By locating and computing the respective peak area percentages using GC post run analysis and EZChrom elite compact software, the fatty acid composition was ascertained (Anjani and Yadav, 2022).

### 2.6 Oil content and oil yield

A seed sample was extracted from each treatment to determine the oil content. The oil content of the seed for each treatment was assessed using a benchtop pulsed nuclear magnetic resonance (NMR) analyser, namely the Oxford MQC-5 model from London, UK. The analyser was equipped with preloaded ‘easy cal’ software and calibrated using known samples of oil groundnut seeds (Tiwari and Burk, 1980). The oil yield (kg ha^-1^) was calculated by the formula:


(1) Oil yield(kg/ha)=Oil per cent in kernel×Kernel yield(kg/ha)

### 2.7 Agronomic-use efficiencies for N and K

Agronomic-use efficiencies [kg pod obtained ÷ N and K fertiliser applied (kg NK/ha^-1^)] were computed with the formulae given by Rana et al. (2014).


(2) $$\begin{array}{c} \text{Agronomic N }\&K\;use\;efficiency\left(\text{kg pod yield/kg }nurtient\;applied\right)\;\\ =\frac{Pod\;yield\;in\;N\&K\;applied\;plots-pod\;yield\;in\;control\left(no-fertilizer\right)plot}{Amount\;of\;nutrient\;N\;and\;K\;applied\left(kg\;ha-1\right)} \end{array}$$

### 2.8 Economic analysis

The economic evaluation involved analysing the costs of cultivation, the net profits obtained, and the benefit:cost ratio (B:C) under different sources of nutrients in groundnut. The cost of executing each treatment was assessed by considering prevailing market prices for inputs and incorporating all expenditures related to the growing of groundnut crop. This included all expenses accrued over the entire process of crop cultivation, combined with shared costs for different operations and resources. The various operations costs were calculated in rupees (Rs.) and then converted to US dollars (US$82 per Rs).

### 2.9 Statistical analysis

The experimental data were analysed statistically using Statistical Software (version 3.2.3) by applying the technique of analysis of variance (ANOVA) prescribed for a split plot design to test the significance of overall difference among treatments by the F test, and conclusions were drawn at a 5% probability level. The effect of treatments was evaluated on a pooled analysis basis of 2 years with regard to growth, yield attributes, yields, and economics. Duncan’s Multiple Range Test (DMRT) was used for the comparison of means.

## 3 Results

### 3.1 Growth and yield attributes

The application of Poly4 fertiliser in conjunction with conventional fertilisers notably increased several yield parameters, such as 100-seed weight, the number of pods per plant, and the shelling percentage of groundnut in two studied locations during 2021–22 and 2022–23 (**Table 2**; **Supplementary Table S1**). However, the application of POLY4 fertiliser did not have a significant impact on the germination rate of groundnut seeds at both locations during the study years. The use of polyhalite fertiliser at the recommended rate of 357 kg ha^-1^, in combination with varying rates of conventional NP fertilisers, resulted in notable improvements in yield attributes like the weight of 100-seed weight (42.1, 39.2, and 40.7 g) and the number of pods per plant (27.3, 24.1, and 25.8) during 2021–22 and 2022–23 and on a pooled basis. However, the application of polyhalite at 357 kg ha^-1^ exhibited a significantly (*P ≤* 0.05) higher shelling percentage of 71.7% (2021–22) and 68.5% (2022–23); these enhancements were particularly evident in plots where a combination of polyhalite and gypsum was applied (T_6_) as compared with plots treated with NPK + gypsum and control plots. However, these yield attributes were at par with the T_5_-polyhalite-applied plots. The control plots (T_10_), which had no fertiliser application, had the lowest yield attributes in both seasons. Among the locations, groundnut grown at ICAR-DGR, Ananthapur, exhibited a significantly higher 100-seed weight (42.0 g) and shelling percentage (67.8%) than the ICAR-CRIDA locations. Conversely, the number of pods was significantly higher (37.2) at the ICAR-CRIDA, Hyderabad, than at other locations.

**Table 2:** The effect of polyhalite-based fertiliser application on pooled growth, yield attributes, and the yield of groundnut at two locations.

Treatment	Germination(%)	100 seed weight (g)	Pods plant^-1^	Shelling %	Pod yield (Mg ha^-1^)	Haulm yield (Mg ha^-1^)	Kernel yield (Mg ha^-1^)	Harvest index (%)	Oil yield (Mg ha^-1^)
Location									
Hyderabad	80.4a	35.4b	37.2a	66.3b	1.79a	2.00b	1.19a	46.5a	0.61a
Ananthapur	79.0b	42.0a	11.0b	67.8a	1.48b	3.52a	1.01b	42.3b	0.49b
Fertiliser sources									
Rec. NPK + gypsum at 500 kg ha^-1^	80.5a	39.7ab	25.2ab	68.2bc	1.76ab	2.82a	1.20abc	46.2ab	0.63a
Rec. NP only	78.4a	37.4d	22.9d	66.0e	1.48e	2.74a	0.98f	41.7d	0.46d
Rec. NPK	80.6a	38.5bcd	24.6abc	66.3de	1.67bcd	2.79a	1.11de	44.8bc	0.56b
Rec. NP + 50% K through MOP	79.8a	38.1cd	23.7cd	66.5cde	1.59d	2.84a	1.06e	42.3cd	0.51c
Rec. NP + 100% K through POLY4	79.7a	40.7a	25.8a	69.7ab	1.83a	2.78a	1.27a	48.2a	0.66a
Rec. NP + 100% K through POLY4 + gypsum at 310	80.2a	40.7a	25.5ab	70.1a	1.76ab	2.74a	1.24ab	47.4ab	0.64a
Rec. NP + 50% K through POLY4	80.2a	39.0bc	24.3bc	67.9bcd	1.71bc	2.83a	1.16bcd	45.2bc	0.58b
Rec. NP + 50% K through POLY4 + gypsum at 310	79.9a	39.6ab	25.1ab	67.6cde	1.69bcd	2.73a	1.14cd	45.5bc	0.58b
Rec. NP + gypsum at 500 kg ha^-1^	80.0a	38.4bcd	23.5cd	65.9e	1.62cd	2.81a	1.07e	44.0cd	0.55bc
Control	77.9a	34.7e	20.9e	62.8f	1.25f	2.51b	0.79g	38.9e	0.35e

*Values followed by the same letter within a column did not differ at the 5% significance level between treatments at DMRT.

**Figure 3:**
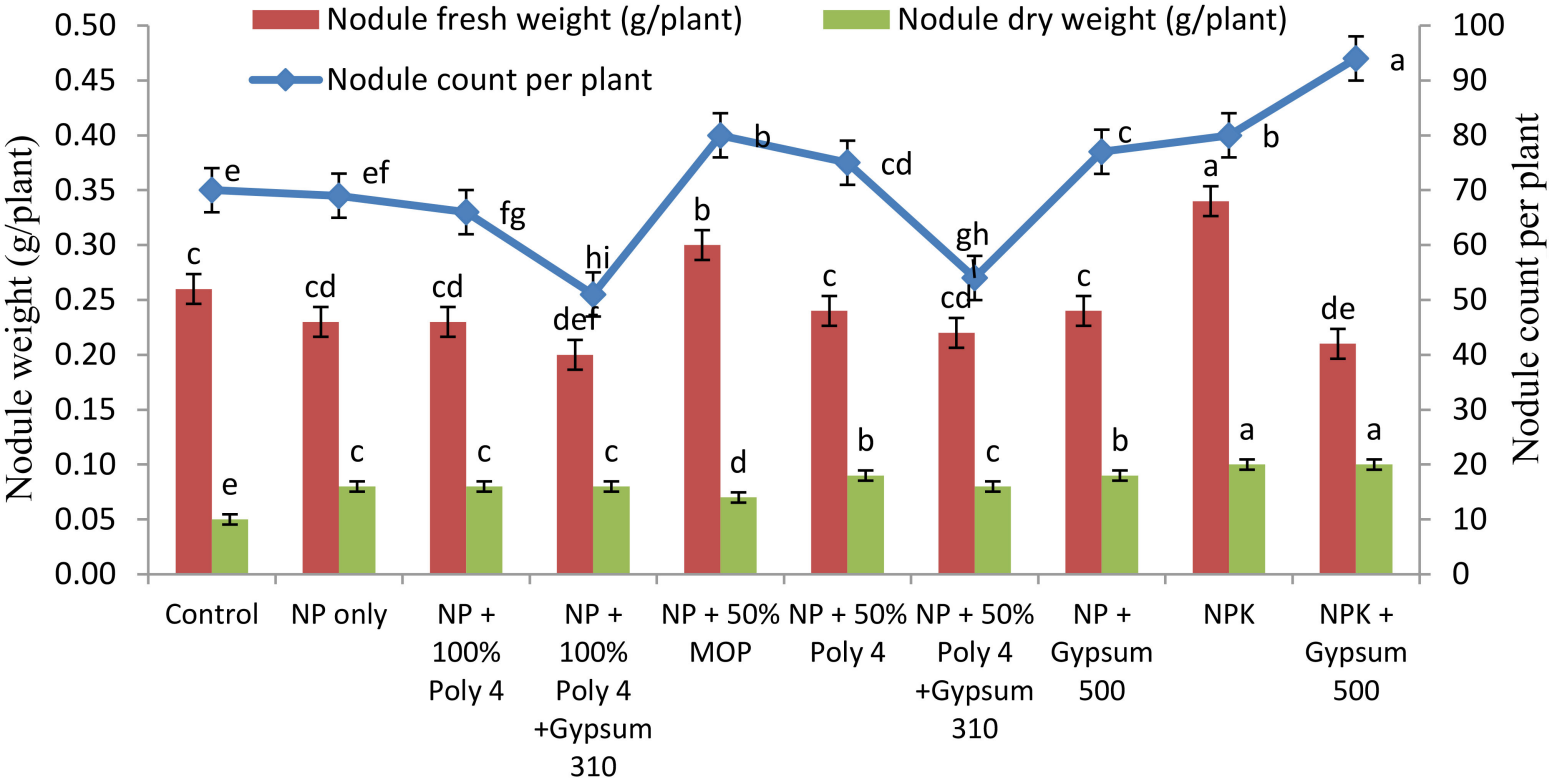
Nodule count and nodule fresh and dry weights in groundnut under POLY4 and other nutrient-source-applied plots during 2022–23. The superscript letters on the bars denote the 5% significance level between treatments at DMRT.

The use of polyhalite fertiliser in conjunction with NP fertilisers had a notable impact on the number of nodules per plant in 2022–23 (**Figure 3**). The application of NPK + gypsum (T_1_) exhibited a significantly (*P ≤* 0.05) higher nodule count (94 per plant) than the other treatments. However, the use of conventional and polyhalite fertilisers, either alone or in combination, did not have a significant effect on the fresh and dry weights of nodules during 2022–23.

### 3.2 Productivity of groundnut

The combined application of POLY4 fertiliser with conventional NP fertilisers applied in different locations had a substantial impact on the pod yield, haulm yield, kernel yield, and harvest index of groundnut in 2021–22 and 2022–23 and on a pooled basis (**Table 2**; **Supplementary Table S2**). The use of POLY4 fertiliser, combined with conventional fertilisers, improved groundnut yield parameters such as seed weight, the number of pods per plant, and shelling percentage at two locations during the 2021–22 and 2022–23 seasons. The application of various rates of conventional fertilisers, in combination with polyhalite fertiliser at 100% of the recommended dose, exhibited an approximately 7.52% higher pod yield than T_1_ and an approximately 29.0% increase in pod yield as compared with control plots during the 2021–22 growing season. In 2022–23, the T_5_ and T_6_ resulted in comparable pod yields of 1.66 and 1.65 Mg ha^-1^, respectively (**Supplementary Figures S3**, **S4**). Overall, 100% polyhalite application exhibited an approximately 4.01% significantly (*P ≤* 0.05) higher pod yield over T_6_. Among locations, groundnut grown at ICAR-CRIDA, Hyderabad, exhibited a significantly (*P ≤* 0.05) higher pod yield (approximately 20.9%) than that at Ananthapur. Likewise, in 2021–22, the plots applied with only NP + 50% K through MOP (T_4_) had a considerably greater pooled haulm yield (2.84 Mg ha^-1^) than the other plots. However, during 2022–23 and on a pooled basis, irrespective of the fertiliser sources, all the fertiliser plots produced a par haulm yield. Similarly, the use of only NP in conjunction with polyhalite fertiliser at rate of 100% resulted in a substantial increase in the kernel yield of 1.42 Mg ha^-1^, 1.13 Mg ha^-1^, and 1.27 Mg ha^-1^, respectively, over other plots during 2021-22 and 2022-23, and on a pooled basis (**Table 2**). The increases in kernel yield in 100% polyhalite applied plots were 10.1, 1.8, and 5.8% over recommended T_1_ during the respective study years. Likewise, T_6_ resulted in par kernel yields when compared with plots treated with 50% polyhalite + NP fertiliser. Therefore, the application of polyhalite fertilisers (T_5_) resulted in a notably (*P ≤* 0.05) higher harvest index (46.2%) than plots treated with conventional fertilisers and control plots throughout the study. Among locations, groundnut grown at ICAR-CRIDA, Hyderabad, exhibited a significantly higher kernel yield (17.8%) than that at ICAR-DGR, Ananthapur. Conversely, ICAR-DGR, Ananthapur, exhibited an approximately 76% higher haulm yield than the other location in the study.

Based on oil content, oil yield also estimated during the study years. Oil yield differed significantly among different fertiliser sources and locations during the study years. Application of 100% polyhalite + NP alone in plots exhibited significantly (*P ≤* 0.05) higher oil yields of 0.75, 0.56, and 0.66 Mg ha^-1^ over other fertiliser-applied plots and control plots during 2021–22 and 2022–23, and on a pooled mean basis, respectively (**Supplementary Table S3**). However, the differences in oil yield under T_5_, T_1_, and T_8_ were on a par with each other. Likewise, groundnut grown at ICAR-CRIDA, Hyderabad, exhibited a significantly higher oil yield (24.5%) than ICAR-DGR, Ananthapur.

### 3.3 Quality attributes

The application of POLY4 fertiliser in combination with conventional fertilisers significantly influenced (*P*<0.05) groundnut quality parameters such as oil content, oleic acid, linoleic acid, palmitic acid, and stearic acid during 2021–22 and 2022–23 and on a pooled mean basis (**Table 3**; **Supplementary Table S3**). The use of POLY4 fertiliser, combined with conventional fertilisers, improved groundnut quality parameters at two locations during the 2021–22 and 2022–23 seasons. The variable rates of conventional fertilisers along with polyhalite fertiliser (T_8_) significantly (*P ≤* 0.05) increased oil content (50.3%), oleic acid (48.7%), palmitic acid (11.1%), and stearic acid (2.88%) compared with other fertiliser sources. However, the difference between T_8_ and T_5_ was found to be at par with each other, whereas the application of the recommended NPK + gypsum at 500 kg ha^-1^ produced a significantly higher linoleic acid (42.1%) content than other fertiliser-applied plots. Interestingly, the inclusion of potassium fertilisers in terms of polyhalite- and MOP-applied plots resulted in a higher oil content and oleic acid content than the application of NP alone and gypsum plots.

**Table 3:** The effect of polyhalite-based fertiliser application on pooled oil content, quality parameters, and the profitability of groundnut at two locations.

Treatment	Oil content (%)	Oleic acid (%)	Linoleic acid (%)	Palmitic acid (%)	Stearic acid (%)	Cost of cultivation (US$ ha^-1^)	Gross returns (US$ ha^-1^)	Net returns (US$ ha^-1^)	B:C
Location									
Hyderabad	50.7a	47.7a	40.7a	11.1a	2.79a	497a	1,279a	749a	1.57a
Ananthapur	48.6b	41.0b	39.4b	10.2b	2.51b	501a	1,125b	624b	1.24b
Fertiliser sources									
Rec. NPK + gypsum at 500 kg ha^-1^	49.9ab	44.5bcd	40.4abcd	10.7ab	2.69abc	505a	1,288ab	783ab	1.55a
Rec. NP only	48.8c	41.4e	42.1a	10.7ab	2.75abc	469a	1,096e	627d	1.34c
Rec. NPK	49.5b	43.9bcd	41.4ab	10.7ab	2.60bcd	477a	1,230bcd	753bc	1.58a
Rec. NP + 50% K through MOP	49.6b	45.1bc	39.1cde	10.3b	2.59bcd	474a	1,174d	700c	1.48ab
Rec. NP + 100% K through POLY4	50.2a	45.4b	40.5abcd	11.1a	2.69abc	534a	1,332a	798a	1.50ab
Rec. NP + 100% K through POLY4 + gypsum at 310	50.3a	48.7a	37.9e	11.1a	2.88a	553a	1,289ab	735abc	1.33c
Rec. NP + 50% K through POLY4	49.5b	44.2bcd	38.4de	10.9	2.78ab	501a	1,254bc	753abc	1.50ab
Rec. NP + 50% K through POLY4 + gypsum at 310	50.2a	44.9bc	39.7bcde	10.9a	2.68abc	521a	1,235bcd	715bc	1.37bc
Rec. NP + gypsum at 500 kg ha^-1^	49.5b	43.1cde	41.2abc	10.4b	2.36d	497a	1,192cd	695cd	1.40bc
Control	48.7c	42.4de	39.4bcde	9.56c	2.50cd	460a	930f	470e	1.03d

*Values followed by the same letter within a column did not differ at 5% significance level between treatments at DMRT.

Among the locations, the growing of groundnut at Hyderabad significantly (*P ≤* 0.05) increased oil content (50.7%), oleic acid (47.7%), linoleic acid (40.7%), palmitic acid (11.1%), and stearic acid (2.79%) compared with Ananthapur. The oil content, oleic acid, linoleic acid, palmitic acid and stearic acid were enhanced by 4.32, 16.3, 3.30, 8.82 and 11.2% under Hyderabad conditions than Ananthapur conditions.

### 3.4 Soil nutrient status

In both locations, fertiliser-applied plots recorded a higher soil organic carbon content and available N, P, and K after the harvest of the groundnut crop than control plots and were found to be on a par with each other (**Table 4**; **Supplementary Tables S4**, **S5**). Likewise, T_1_ had significantly (*P ≤* 0.05) higher SOC (0.42%), available N in the soil (127.7 mg kg^-1^), available P (29.6 mg kg^-1^), and available K (152.3 mg kg^-1^) than other fertiliser-applied plots. However, polyhalite and gypsum application alone or in combination also retained higher soil macronutrients than control plots. Among the locations, groundnut grown in Hyderabad maintained higher SOC (0.47%), available N (130.4 mg kg^-1^), and available P (41.1 mg kg^-1^) than Ananthapur. However, the application of polyhalite fertiliser with conventional NP exhibited available SOC and NPK that was on a par with the recommended NPK + gypsum-applied plots. Similarly, polyhalite-applied plots retained more soil micronutrients such as Cu, Fe, Zn, and Mn as than gypsum and MOP-applied plots (**Table 4**). However, adding fertilisers with or without polyhalite did not significantly change the amount of copper and zinc in the soil. However, the application of 100% POLY4 fertiliser in combination with conventional NP fertilisers significantly increased soil manganese (32.3 mg kg^-1^) compared with other treatments. Groundnut grown in Ananthapur exhibited higher Fe (22.3 mg ka^-1^), Zn (1.38 mg ka^-1^), and Mn (40.6 mg ka^-1^) than in Hyderabad.

**Table 4:** The effect of polyhalite-based fertiliser application on soil organic carbon (SOC) and the soil available nutrient status after harvesting of groundnut at two locations.

Treatment	SOC (%)	Available N (mg kg^-1^)	Available P (mg kg^-1^)	Available K (mg kg^-1^)	Cu (mg kg^-1^)	Fe (mg kg^-1^)	Zn (mg kg^-1^)	Mn (mg kg^-1^)
Location								
Hyderabad	0.47a	130.4a	41.1a	123.3b	1.62a	8.63b	0.46b	21.1b
Ananthapur	0.34b	117.9b	13.9b	163.5a	0.89b	22.3a	1.38a	40.6a
Fertiliser sources								
Rec. NPK + gypsum at 500 kg ha^-1^	0.42a	127.7a	29.6a	152.3a	1.29ab	16.1ab	1.05a	29.5bc
Rec. NP only	0.39de	119.4b	26.3e	133.6e	1.24ab	14.6ef	0.88bcd	32.4a
Rec. NPK	0.40cd	125.9a	28.8ab	147.1bcd	1.18b	15.1def	0.94bc	30.9ab
Rec. NP + 50% K through MOP	0.40bcd	120.6b	27.3cde	143.3d	1.17b	15.3cde	0.82d	30.7ab
Rec. NP + 100% K through POLY4	0.41abc	126.5a	28.4abc	151.3ab	1.37a	15.9abc	0.97ab	32.3a
Rec. NP + 100% K through POLY4 + gypsum at 310	0.42ab	127.9a	28.7ab	150.4ab	1.24ab	16.0abc	1.05a	29.5bc
Rec. NP + 50% K through POLY4	0.41abc	126.6a	26.9de	148.4abc	1.21b	16.3a	0.95ab	29.7bc
Rec. NP + 50% K through POLY4 + gypsum at 310	0.41abc	124.8a	28.0bcd	144.8cd	1.23ab	15.4bcde	0.90bcd	32.4a
Rec. NP + gypsum at 500 kg ha^-1^	0.40bcd	127.3a	28.6abc	137.1e	1.33ab	15.5abcd	0.85cd	33.0a
Control	0.38e	115.0c	24.1f	125.8f	1.25ab	14.4f	0.80d	27.9c

*Values followed by the same letter within a column did not differ at the 5% significance level between treatments at DMRT.

### 3.5 The agronomic efficiency of N and K

**Figure 4:**
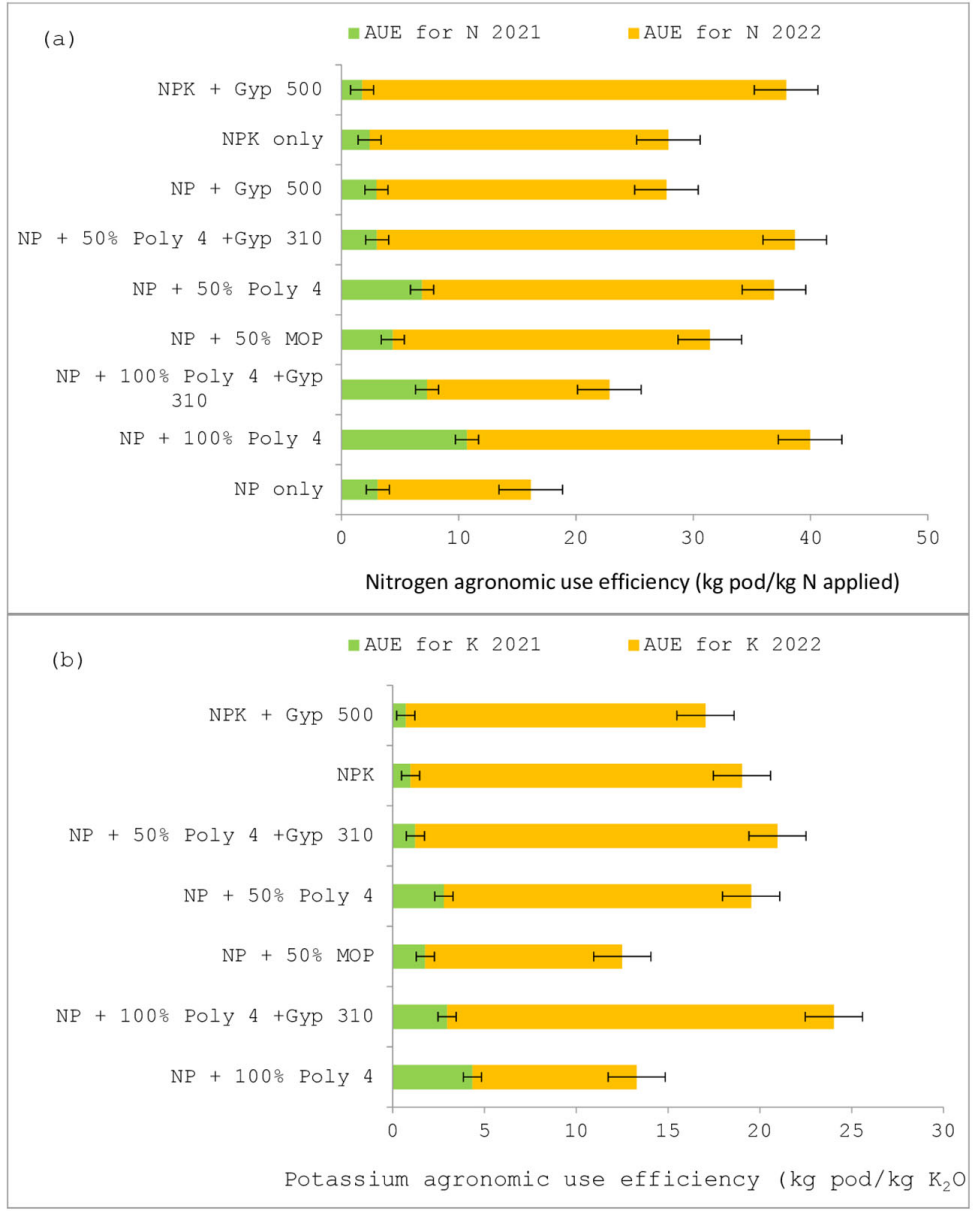
The agronomic use efficiency of **(A)** nitrogen and **(B)** potassium in groundnut under POLY4 and other nutrient-source-applied plots during 2021–22 and 2022–23.

In terms of the agronomic efficiency (AE) of N, the application of the recommended NP + 100% POLY4 (T_5_) increased the AE for N during 2021–22, a period of higher drought stress, by 10.7 kg pod kg^-1^ N applied as compared with other plots (**Figure 4**). During 2022–23, the application of NPK fertilisers along with gypsum produced 36.2 kg pod kg^-1^ N applied more than in other plots. However, it was on a par with T_8_ (35.6 kg pod kg^-1^ N applied). The application of NP fertilisers only exhibited a significantly lower AE of N than other plots.

Likewise, the application of recommended NP + 100% POLY4 (T_5_) increased the AE for K up to 4.35 kg pod kg^-1^ K_2_O applied during 2021–22 as compared with other plots (**Figure 4**). During 2022–23, the application of NP + 100% POLY4 + gypsum at 310 kg ha^-1^ produced 21.07 kg/pod kg^-1^ K_2_O applied more than in other plots. However, it was on a par with T_8_ (19.73 kg/pod kg^-1^ K_2_O applied). The application of NP fertilisers + 50% MOP only resulted in a significantly lower AE of K_2_O than other plots.

### 3.6 Profitability

The use of polyhalite fertiliser in conjunction with traditional fertilisers had a substantial impact on the profitability of groundnut cultivation in both the studied locations during 2021–22 and 2022–23 (**Table 3**; **Supplementary Table S6**). The use of varying rates of standard NP fertilisers, in combination with polyhalite fertiliser at 100% + gypsum at a rate of 310 kg ha^-1^, resulted in increased costs of cultivation (COC) of US$551, 555, and 553 per hectare during the years 2021–22 and 2022–23, and on a pooled basis, respectively (**Supplementary Table S6**). The use of polyhalite at full rates in conjunction with NP fertilisers resulted in slightly reduced COC of US$532, 536, and 534 per hectare compared with plots where gypsum was used.

The application of 100% polyhalite + NP fertilisers resulted in notably (*P ≤* 0.05) greater pooled gross returns (US$1,332 ha^-1^) and net returns (Rs. 798 ha^-1^) than the other plots. The use of polyhalite (100%) + NP fertilisers resulted in a significant (P ≤ 0.05) increase in gross returns (4.85%) and net returns (5.77%), as compared with T_1_ (**Table 3**). Likewise, the use of 50% POLY4-applied plots resulted in slightly reduced net returns, but which were statistically comparable with those of T_1_. Nevertheless, the analysis revealed that the pooled benefit:cost ratio (B:C) differed statistically and it was higher with the application of recommended NPK + gypsum T_1_ (1.55)-applied plots than others but it was on a par with polyhalite-applied plots. In general, the use of polyhalite at either 100 or 50% demonstrated a notable increase in net returns, ranging from US$45 to US$50 with the same level of management practices, when compared with the application of NPK fertilisers only.

## 4 Discussion

The effective adoption of crop nutrition is of utmost importance, especially in arid regions of Alfisols in rainfed situations. The soil composition mostly consists of sandy particles and exhibits a significantly low cation exchange capacity (CEC), which limits its ability to retain and store nutrients. Additionally, the pH level of the soil is rather low (pH 5.6–6.1), further diminishing its capacity to retain essential nutrients. Simultaneously, to effectively utilise gypsum for the provision of calcium (Ca) and sulphur (S) during the flowering stage, it is essential to provide adequate moisture levels. Therefore, sufficient moisture is crucial for maximising the benefits of gypsum application in rainfed groundnut cultivation. In the current investigation, the application of a gypsum supplement (500 kg ha^–1^) along with the recommended NPK fertilisers resulted in lower pod and kernel yields of groundnut than those with polyhalite fertiliser application (**Table 2**). The pod yield increases were approximately 7.5–29.0% greater than those of gypsum applied plots and control plots. The increased crop output from using polyhalite may be attributed to the synchronised and gradual release of nutrients, which allows plants more time to absorb the available nutrients. Potential reasons for the decreased production in plots where gypsum was applied may include reduced moisture availability during drought stress, gypsum having less solubility at the time of application, and a failure to increase the growth of groundnut plants. Similarly, when gypsum and polyhalite are applied together, the resulting nutrient balance might not align well with the nutritional requirements of peanut. Excess calcium from gypsum can interfere with the uptake of other nutrients provided by polyhalite, particularly potassium and magnesium, which are critical for peanut growth (Marschner, 2012; Singh et al., 2023). Likewise, gypsum and polyhalite release sulphate ions into the soil. Excessive sulphate from both sources could potentially lead to competition among anions for uptake by plants, affecting nutrient availability and plant growth (Cardoso et al., 2022). The potential advantages of gypsum application at the prescribed timeframe of 25 days after sowing (DAS) were not immediately visible due to the influence of drought-induced stress experienced during that specific duration.

However, in the current study, the application of polyhalite fertiliser at a rate of 100% along with only NP fertilisers resulted in a significant (*P ≤* 0.05) increase in pod yield (5.3–7.5%) over NPK + gypsum and control plots. It seems that adding polyhalite had a noticeable effect on the groundnut yield attributes and yield. Kumar et al. (2023) opined that the application of polyhalite fertiliser increases oat growth and improves yield attributes. A possible reason for this may be the gradual release of nutrients provided by the polyhalite fertiliser. Polyhalite’s primary mode of nutrient release is through its dissolution in soil moisture. This slow-release process helps in providing a steady supply of these essential nutrients to crops over an extended period, reducing the risk of nutrient leaching (Tan et al., 2022). Furthermore, in acidic soils, the dissolution rate is higher due to the presence of hydrogen ions that can facilitate the breakdown of polyhalite (Singh et al., 2023). This also links the probable reason for the poor germination rate of groundnut seeds, indicating that POLY4 enhances yield attributes during later growth stages. This observation also aligns with the findings of Zhang et al. (2018), who also reported no significant impact of polyhalite on seed germination rates. Therefore, the nutrient delivery capability is not hindered by the presence of moisture, as polyhalite exhibits delayed solubility during the crop growth stages under conditions of drought stress. Thus, the application of polyhalite resulted in a significantly (*P ≤* 0.05) higher groundnut yield (5.3–7.5%). The observed increase in crop output can perhaps be attributed to the greater average weight of 100 seeds and the improved shelling percentage in the plots treated with polyhalite. Similarly, other studies have provided evidence for the effectiveness of polyhalite in increasing crop output, particularly in rice (Huang and Chen, 1990), corn (Wang et al., 2018), wheat (Kumar et al., 2023), oat (Ola et al., 2023) peanut (Tan et al., 2022), potato (Mello et al., 2018a), mustard (Tiwari et al., 2015), tomato (Mello et al.), and sugarcane (Bhatt et al., 2021; Herrera et al., 2022). Thus, the increase in yield in polyhalite-applied plots was found to enhance the quality parameters such as oil (50.3%), oleic acid (48.7%), palmitic acid (11.1%), and stearic acid (2.88%) content in groundnut (**Table 4**). Interestingly, the inclusion of potassium fertilisers in terms of polyhalite and MOP in the treatments resulted in a higher oil content, oil yield, and oleic acid content than the application of NP alone and gypsum plots, whereas, the application of polyhalite fertiliser (100% recommended) + NP + gypsum at 310 kg ha^-1^ reduced the palmitic acid (11.01%) content in the groundnut seed compared with other plots.

Among the locations, groundnut grown at ICAR-CRIDA, Hyderabad, exhibited a significantly (*P ≤* 0.05) higher pod yield (approximately 20.9%) than groundnut grown at Ananthapur due to better rainfall conditions and higher SOC (0.47%), available N (130.4 mg kg^-1^), and available P (41.1 mg kg^-1^) than the other location. According to Miller et al. (2022), this variation could be attributed to location-specific factors such as soil type, climate conditions, and inherent fertility, which are known to influence crop responses to fertilisation. Concurrently, throughout the duration of the trial, plots treated with a combination of polyhalite and gypsum exhibited yields that were on a par with those at plots treated with either polyhalite or gypsum. However, during 2022–23, the recommended fertiliser-applied plots (NPK + gypsum) produced much greater haulm yields than the other treatments. Although these plots exhibited a notable increase in haulm production, they were unable to effectively convert the accumulated source into sink. According to the findings of Huang and Chen (1990), polyhalite demonstrates a greater suitability for utilisation in areas characterised by high levels of precipitation and a limited availability of potassium (K) due to its gradual release properties. Therefore, the application of polyhalite tends to increase agronomic use efficiency (AE) for N and K_2_O as compared with others (**Figure 4**).

Fertiliser-applied plots recorded higher soil organic carbon content and available P and K after the harvest of groundnut crop than control plots. Likewise, the application of NPK + gypsum at 500 kg/ha in plots resulted in significantly higher SOC (0.42%), available N (127.7 mg kg^-1^), available P (29.6 mg kg^-1^), and available K (152.3 mg kg^-1^) than others (**Table 4**). However, the application of polyhalite fertiliser with conventional NP resulted in available N levels that were on a par with NPK + gypsum-applied plots. However, fertiliser addition with or without polyhalite did not significantly change the amount of copper and zinc in the soil. Therefore, the availability of potassium (K), calcium (Ca), magnesium (Mg), and sulphur (S) is crucial for the proper growth and development of peanut plants (Trankner et al., 2018; Tan et al., 2022). This, in turn, facilitates efficient nitrogen fixation by the peanut roots, leading to the enrichment of soil with nitrogen. The insufficiency of calcium negatively impacts the process of photosynthate translocation and dispersion, as well as the development of pods and overall crop yield (Dang et al., 2018). Zheng et al. (2018) and Tan et al. (2022) opined that magnesium (Mg) serves as a catalyst for multiple enzymes and assumes crucial functions in the synthesis of carbohydrates, proteins, and lipids. According to Wang et al. (2017), fertilisation K and Ca had a substantial impact on the accumulation of nutrients in peanut stem leaves. The application of polyhalite fertiliser facilitated the transportation of K, Ca, and Mg to the pods, thereby leading to the cultivation of peanuts of superior quality (Ma et al., 2021). Conversely, polyhalite was used as a partial alternative due to its limited availability of Ca and Mg. When the concentrations of Ca and Mg are relatively low, the interplay between K, Ca, and Mg primarily manifests as a synergistic response (Wang et al., 2021).

The fundamental objective of peanut production is to increase the yield and financial profitability. In the current study, the use of polyhalite fertiliser in conjunction with traditional fertilisers had a substantial impact on the profitability of groundnut cultivation in the years 2021–22 and 2022–23 (**Table 3**). The use of polyhalite (100%) + NP fertilisers resulted in a significant increase in gross returns (4.85%) and net returns (5.77%) compared with plots treated with NPK + gypsum (T_1_). When compared with applying only NPK fertilisers, the use of polyhalite at either 100% or 50% generally resulted in a noticeable improvement in net yields, ranging from US$45 to US$50 per hectare. The increased groundnut yields in the corresponding treatments that used polyhalite fertilisers at optimal rates may be responsible for the observed increase in net returns. Similarly, the relatively lower potassium (K) percentage of polyhalite fertiliser lowers its cost (Singh et al., 2023). The primary factors influencing net revenue are variations in peanut production and expenses related to K fertiliser. Therefore, the implementation of polyhalite yielded the maximum amount of product and generated the greatest financial gain. This outcome is advantageous for mitigating the challenges posed by the scarcity of potash resources.

## 5 Conclusion

The application of various quantities of polyhalite fertiliser as a substitute for muriate of potash and gypsum fertiliser has demonstrated significant improvements in plant growth and yield, soil chemical characteristics, and economic benefits as a whole. The application of polyhalite (Poly 4) along with NP fertilisers resulted in an approximate 5.3–7.5% yield advantage over NPK + gypsum-applied plots during the study years. The application of polyhalite fertiliser (100% recommended) significantly increased pod yield by 12.8 and 5.3% in NP + 100% polyhalite-applied plots compared with NPK + gypsum (500 kg ha^-1^) and control plots, respectively. The key factor that exhibited higher yields was the supply of a combination of four essential nutrients, potassium (K), calcium (Ca), magnesium (Mg), and sulphur (S), collectively with a higher solubility and supply of nutrients. The application of potassium through polyhalite and MOP increased oleic acid content in groundnut over NP fertilisers only and *vice versa* in terms of linolic acid content. Additionally, the profits obtained from the polyhalite fertiliser substitution treatment were found to be the highest among the treatments. An increase in gross returns of 4.85% and in net returns of 5.77% were recorded with the application of polyhalite (100%) + NP fertilisers. Hence, the application of a 100% polyhalite fertiliser or a 50% polyhalite blend emerges as a viable option to augment peanut crop yield and foster agricultural sustainability. Therefore, the integration of polyhalite in groundnut fertilisation practices not only augments crop yield and quality but also offers significant economic advantages, thus providing a robust alternative to traditional fertilisation methods and contributing to the resilience and sustainability of agricultural systems.

## Data Availability

The original contributions presented in the study are included in the article/**Supplementary Material**. Further inquiries can be directed to the corresponding authors.
